# Skin Affections Reflecting Internal Maladies*Read before the “Maimonides Club” Nov. 1917.

**Published:** 1918-02

**Authors:** Jos. Spangenthal

**Affiliations:** Buffalo


### Skin Affections Reflecting Internal Maladies.*

By JOS. SPANGENTHAL, M. D., Buffalo.

Too often does the internist consider the Dermatologist as a specialist distinctly isolated in his chosen specialty, bearing no relationship to the other branches of their profession. The Dermatologist should be broad in his knowledge of general pathology involving internal diseases, and should become familiar with their diagnosis and treatment. Likewise should the Internist be acquainted in a superficial way at least, with the multitude of skin affections which reflect some internal disease.

This inter-relation could be more easily comprehended, if we would for a moment consider the skin as an essential organ, analogous to the lungs and the kidneys, whose physiological function is somewhat similar. Like the other organs, it is composed of connective tissue, lymphatics, nerves, blood-vessels, etc., and is subject to the same pathological changes. In the importance of its physiological function, it may be comparable to the kidneys. It excretes waste material in the form of urea and other poisonous substances, and exhales noxious gases like the lungs. This same analogy should be recognized in its pathology. We will now consider some of these skin affections.

ROSACEA: This is a quite common disease occurring in both sexes, in adult life, presenting itself on the cheeks, chin, nose, and forehead, in patches of hyperaemia in which are observed the permanently dilated capillaries. This disease is often associated in the female with uterine or gastric disorder, usually of a functional nature. In the male, Rosacea may develop into Rhinophyma, lobulated enlargement of the nose, concomitant with catarrhal gastritis occasioned by long continued over-indulgence in alcoholics.

CARBUNCLE: The knowledge that carbuncle and diabetes are so often associated in the same individual, should make one alert to examine the urine for glucose in every case presenting a carbuncle.

CHLOASMA: The term applied to increased pigmentation of the skin occurring as variously sized and shaped yellowish, brownish, or blackish patches, or more or less diffused discoloration. The symptomatic variety is observed in association with tuberculosis, sarcoma, cancer, malaria, Addison's disease, Graves' disease, and functional and organic diseases of the utero-ovarian system. Under this classification

*Read before the "Maimonides Club" Nov. 1917.

might be mentioned the greenish hue of chlorosis, the lemon tint of pernicious anaemia, the straw color of carcinoma, and the waxy pallor of parenchymatous nephritis.

ANIDROSIS: A functional disorder of the sweat glands characterized by diminution or suppression of the sweat secretion. This is rarely an idiopathic condition, but it occurs in certain systemic diseases especially diabetes.

ERYTHEMA NODOSUM: This is an inflammatory affection of an acute type, characterized by the formation of variously sized roundish, more or less elevated, erythematous nodes or swellings, attended with some degree of systemic disturbance. The frequently associated rheumatic symptoms observed would indicate some connection with this disease, and Curshman states that in 25 cases he met with hemorrhagic nephritis 5 times.

ERYTHEMA MULTIFORME: This may be defined as an inflammatory disease of an acute character ushered in by reddish or purplish red, often variegated macules, papules or tubercles, occasionally becoming vesicular or bullous. This disease is probably of an infectious nature, and there are not infrequently associated rheumatic symptoms; in fact articular rheumatism may follow an attack of this disease. Apropos, the following case will illustrate this. W. E., aet. 30, was taken ill with acute follicular tonsillitis ushered in with high temperature and systemic disturbance. The following day his face, arms, and trunk were covered with eruption of erythema multiforme, of the erythematous and papular variety. After a week's duration, the eruption disappeared, and he then suffered from a severe attack of acute articular rheumatism, in which the knee and wrist joints were involved.

DERMATITIS FACTITIA, or feigned skin eruptions. These cases are of interest, because of their appearance upon patients, usually females of a neurotic tendency, showing hysterical symptoms. They may simulate almost any skin lesion, and are often very difficult of diagnosis. Their importance in indicating some instability of the nervous system should be considered.

FURUNCULOSIS: Two factors are to be considered in this disease, essential and predisposing. Among the many predisposing causes, may be mentioned nephritis and diabetes mellitus.

HERPES ZOSTER: This disease, acutely inflammatory in nature, is characterized by the appearance of several or numerous groups of vesicles on an inflamed area, of unilateral distribution, corresponding to some cutaneous nerve. There is also present more or less neuralgic pain, sometimes of great intensity. This pain often precedes the eruption by several days, and is due to an acute neuritis, thus exemplifying the relation between a dermatological lesion and a nerve involvement.

HYPERIDROSIS: Excessive amount of sweating in a general form, may be symptomatic of some underlying unrecognized disease such as incipient Graves' disease or tuberculosis.

NAILS: The nails as an appendage of the skin, often by their deformity as well as the diseases which affect their substance, indicate serious constitutional maladies. We are all familiar with the clubbing of the nails which is suspicious of tuberculosis. When in addition, the nails are cyanotic, we invariably find cardiac affection of congenital origin.

HAIR: Alopecia Areata and Alopecia Syphilitica are the two forms that indicate some constitutional disturbance, the former being often of neurotic origin, the latter one of the early manifestations of luetic infection.

Apropos of alopecia areata neurotica, the following case observed by the writer will be of interest. A young adult who had been under a severe business strain, suffered from this disease. Within the period of several weeks, the hair from his head, beard, eye-brows, lashes, axillae, and pubis was lost. This condition persisted for two years despite the vigorous treatment that had been applied over a period of 6 months. Several months ago the patient spent 6 weeks' vacation on a boat trip. Upon his return, he exhibited a complete recovery. This furnished strong corroboration of the neurotic theory of this disease.

PELLAGRA: This disease, of such frequency in Italy, and in our southern states, usually comes under the observation of the dermatologist. The skin manifestations for the most part appear upon the backs of the hands and forearms in the nature of a dermatitis, but the constitutional aspect is of such importance, that the skin affection per se would seem of secondary consideration. The constitutional symptoms which stand out most prominently are, malassimilation with depression, or even a psychosis in the nature of a severe melancholia with delusions. There are also present gastroenteric symptoms of great severity.

PEMPHIGUS: An acute or chronic bullous disease of the skin, characterized by the formation of scanty or numerous variously sized blebs, which may be accompanied by mild or severe constitutional symptoms. The etiology of pemphigus is obscure, and the prognosis often grave. I mention this malady to show the relation that exists between the skin lesion and the serious systemic infection.

PRURITUS: A functional cutaneous affection, manifesting itself solely by the presence of itching without structural alteration of the skin. Pruritus may be a distinct affection, or it may signify some other malady of which it forms one of its symptoms. The most common factors to be considered as of probable import in generalized pruritus are digestive and intestinal derangement, hepatic disorders, intestinal worms, Bright's disease, diabetes mellitus, ovarian and uterine disorders, carcinoma, and a depraved state of the nervous system. Also certain drugs, especially opium should be considered.

PURPURA: A hemorrhagic affection of the skin in which appear variously sized smooth, reddish or purplish spots or patches, not disappearing upon pressure, and accompanied by systemic disturbance of slight or severe nature. In considering the etiological factors in general, this disease may be classified as vasomotor, toxic and infectious. Those of toxic origin arise from an auto-intoxication starting in the intestinal tract, thus indicating the close relation existing between this disease and some faulty metabolism.

EXANTHEMATA: Scarlatina, Rubeola, Varicella, and Variola are constitutional diseases of infectious nature, in all of which are skin manifestations, again showing the organic correlation.

URTICARIA: The indirect or internal causes of urticaria are numerous, most of them acting through the alimentary tract. Emotional or psychic causes such as anger, fright or sudden grief will sometimes occasion an outbreak, demonstrating the effect of the sympathetic nervous system upon the skin.

XANTHOMA: A slightly elevated, flattened, or somewhat rounded, soft neo-plastic growth, of a yellowish color, usually seated as one or several lesions about the eye-lids, and occasionally of more or less general distribution. Among the etiological factors or rather associated with this disease may be mentioned jaundice; and Kaposi, Hardaway, and others are inclined to believe that the xanthoma growths are also developed in the liver.

In presenting these various skin affections, I have by no means exhausted the subject. There are many diseases of the skin of probable internal origin whose etiology is yet obscure. Among these may be mentioned Dermatitis Herpetiformis, Lichen Planus, Psoriasis, some of the Eczemas, Epidermolysis Bullosum, etc.

I hope the point has been made clear, that there is constantly a correlation between that most important organ the skin, and all of the other organs.

595 Lafayette Ave.

---

**Traumatisms and Morbidity in Troops.** An anonyomus surgeon, Western Med. Rev., Oct., gives the following estimate and results of sanitary and hygienic training:

Table of Probable Number of Cases of Disability in an Army of 1,000,000 Men.

| Diseases: | Total Cases | Cases Per 1,000 |
|---|---|---|
| Typhoid fever | 800 | 0.80 |
| Paratyphoid | 14,680 | 14.68 |
| Measles | 19,930 | 19.93 |
| Scarlet fever | 130 | 0.13 |
| Influenza | 21,340 | 21.34 |
| Tuberculosis | 3,890 | 3.89 |
| Insanity | 1,680 | 1.68 |
| Appendicitis | 5,990 | 5.99 |
| External causes: | | |
| Fractures | 11,530 | 11.53 |
| Dislocations | 3,150 | 3.15 |
| Gunshot wounds | 1,970 | 1.97 |
| Sprains | 45,980 | 45.98 |
| Wounds, except gunshot | 33,950 | 33.95 |
| Other external causes | 65,390 | 65.39 |
| Total all causes | 887,800 | 887.80 |

---

**Sunlight in the Treatment of Wounds.** D. Freche, Jour. de Med. de Bordeaux, mentions this as a favorite at present. He concedes the bactericidal power, approximately 96% of which is due to violet and blue rays, 4% to rays at the lower end of the spectrum (calorific). But, for anthrax spores, 29-54 hours, exposure are necessary and even 83 hours if protected from the air. The bactericidal power is diminished by the presence of moisture—inevitably present in wounds,—by haemoglobin which absorbs the most active rays, and is very slight beneath the surface. Irradiation is also painful in many cases. Sunlight has a special indication in atonic wounds but even here other methods, physical or chemic, are superior. On the whole, he believes the advocates of the method are the victims of a mirage of their own creation.

